# The Beneficial Effect of Boswellic Acid on Bone Metabolism and Possible Mechanisms of Action in Experimental Osteoporosis

**DOI:** 10.3390/nu12103186

**Published:** 2020-10-18

**Authors:** Bandar E. Al-Dhubiab, Snehal S. Patel, Mohamed A. Morsy, Harika Duvva, Anroop B. Nair, Pran Kishore Deb, Jigar Shah

**Affiliations:** 1 Department of Pharmaceutical Sciences, College of Clinical Pharmacy, King Faisal University, Al-Ahsa 31982, Saudi Arabia; momorsy@kfu.edu.sa (M.A.M.); anair@kfu.edu.sa (A.B.N.); 2 Department of Pharmacology, Institute of Pharmacy, Nirma University, Ahmedabad 382481, Gujarat, India; snehalpharma53@gmail.com (S.S.P.); 11mph205@nirmauni.ac.in (H.D.); 3 Department of Pharmacology, Faculty of Medicine, Minia University, El-Minia 61511, Egypt; 4 Department of Pharmaceutical Sciences, Faculty of Pharmacy, Philadelphia University, Amman 19392, Jordan; prankishore1@gmail.com; 5 Department of Pharmaceutics, Institute of Pharmacy, Nirma University, Ahmedabad 382481, Gujarat, India; jigsh12@gmail.com

**Keywords:** acetyl-11-keto-β-boswellic acid, osteoporosis, ovariectomy, NF-κB, TNF-α

## Abstract

Estrogen is instrumental in the pathological process of osteoporosis because a deficiency of this hormone increases the release of bone-resorbing cytokines. Acetyl-11-keto-β-boswellic acid (AKBA), a constituent from *Boswellia serrata*, has an anti-inflammatory effect by inhibiting tumor necrosis factor-α (TNF-α) expression, which leads to a decline in receptor activator of nuclear factor-kappa B (NF-κB) ligand, and consequently, a reduction in osteoclast activity. Hence, AKBA may be beneficial against bone loss during osteoporosis. Therefore, the current study intended to evaluate the beneficial effects of AKBA in ovariectomy-induced osteoporosis and to investigate its mechanism of action. Sham-operation or ovariectomy female Sprague Dawley rats were used for evaluating the antiosteoporotic effect of AKBA in this study. AKBA (35 mg/kg, p.o.) and estradiol (0.05 mg/kg, i.m.) were administered for 42 days. At the end of the experiment, body and uterus weights, serum and urine calcium and phosphorus, serum alkaline phosphatase, and urinary creatinine levels, besides serum levels of NF-κB and TNF-α were determined. Weight, length, thickness, hardness, calcium content, as well as the bone mineral density of femur bone and lumbar vertebra were measured. A histopathological examination was also carried out. AKBA ameliorated all tested parameters and restored a normal histological structure. Thus, AKBA showed good antiosteoporotic activity, which may be mediated through its suppression of the NF-κB-induced TNF-α signaling pathway.

## 1. Introduction

Osteoporosis, a pandemic health issue affecting generally postmenopausal women, is a systemic skeletal disease known with decreased bone mineral density (BMD) and micro-architectural impairment of the quality of bone tissue [1]. This debilitating condition of osteoporosis leads to a higher risk of bone brittleness and vulnerability to fracture and is associated with morbidity, mortality, and a reduction in quality of life [2]. Estrogen plays a pivotal role in the pathological process of postmenopausal osteoporosis because the deficiency of this chief hormone increases the release of bone-resorbing cytokines, which appears to increase the osteoclast activity [3]. Currently, anti-resorptive and anabolic agents are extensively used for the treatment of osteoporosis. However, highly efficacious agents with outstanding safety and efficacy are necessary. Extensive studies has been carried out in the past few decades to understand osteoblast differentiation and bone formation, and the drug discovery research continued their effort to develop new therapeutic agents that can prevent and/or treat bone diseases [4,5,6]. Further, previous studies suggested that metabolic dysfunction and osteoporosis share common pathways, which include the regulation of calcium homeostasis, receptor activator of nuclear factor-kappa B (NF-κB) ligand (RANKL), osteoprotegerin, and Wnt-β-catenin signaling pathways [7]. Osteoblasts synthesize RANKL, which is a molecule from the tumor necrosis factor (TNF) superfamily, necessary for osteoclast formation as well as function. RANKL receptors are key regulators of bone remodeling. RANKL produces the variation of osteoclast precursor cells and activates the selective uptake function and survival of developed osteoclasts. Estrogen, which is reported to impede the expression of RANKL, plays a potential role in bone remodeling [8,9]. Thus, RANKL signaling axis plays a critical role in the process of bone formation.

**Figure 1:**
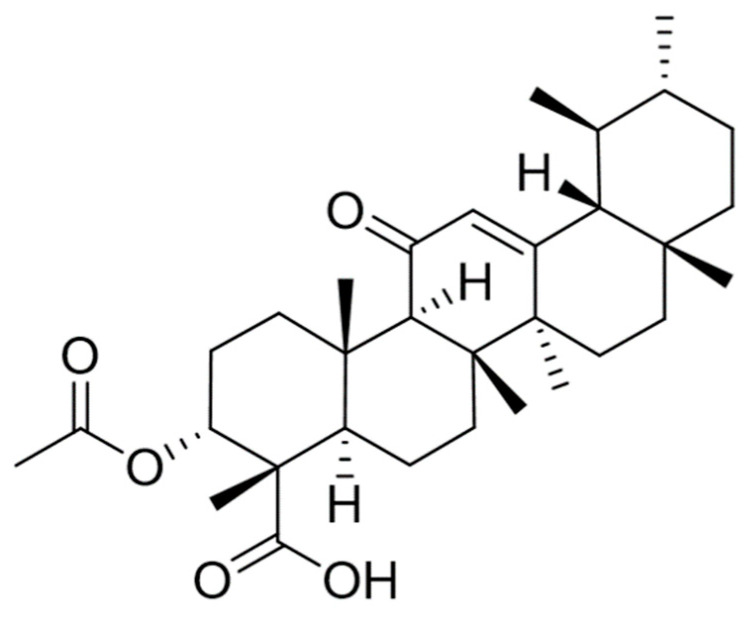
Chemical structure of acetyl-11-keto-β-boswellic acid.

*Boswellia serrata* is a small branching tree usually found in dry lands of Southern Asia, North Africa, as well as in Arab countries. Traditionally *Boswellia serrata* has been considered as an Ayurvedic medicine since antiquity for the treatment of various disorders. Barks of *Boswellia serrata* are rich in gummy oleo resin, oils, terpenoids, sugars, and volatile oils [10]. The boswellic acid is an important constituent separated from the oleo-gum resin of *Boswellia serrata*, consisting of pentacyclic triterpenic acids. Indeed, twelve or more boswellic acids have been recognized and characterized in the gum resin. Among these only 11-keto-β-boswellic acid and acetyl-11-keto-β-boswellic acid (AKBA) (Figure 1) received significant interest owing to their various therapeutic activities [11]. Boswellic acid has been reported to produce anti-inflammatory, antiarthritic, antirheumatic, antidiarrheal, antihyperlipidemic, antiasthmatic, anticancer, antibacterial, antifungal, anticomplementary, and analgesic activity [10,12,13,14,15].

The treatment of osteoporosis typically promotes osteoblasts differentiation, which subsequently results in bone formation [16]. A review of literature signifies that inhibition of NF-κB expression leads to a decline in RANKL expression, resulting in osteoblast differentiation and consequently reducing bone loss [17,18]. Previous studies reported that boswellic acid has a positive impact in the treatment of chronic inflammatory diseases by different mechanisms of action [19,20]. In addition, the anti-inflammatory actions of AKBA are due to the inhibition of leukotriene synthesis and a decrease in the formation of proinflammatory cytokines, which includes interleukin (IL)-1, IL-2, IL-6, interferon-γ, TNF-α, and NF-κB, which are primarily responsible for destroying bone cartilage. Among all these, NF-κB is believed to be the potential target for AKBA [14,21]. Further, in vitro and in vivo data demonstrated that AKBA inhibits osteoclastogenesis by hindering NF-κB and NF-κB-regulated gene expression [22] and has a positive impact in the prevention as well as treatment of periprosthetic osteolysis [23]. Therefore, the objective of our study was to assess the antiosteoporotic effect of AKBA in an ovariectomized in vivo model of osteoporosis and to investigate its mechanism of action. The effect of AKBA was evaluated by various physical and biochemical (serum/urine) parameters, NF-κB and TNF-α expressions, and histopathological changes in ovariectomized rats.

## 2. Materials and Methods

### 2.1. Chemicals and Kits

AKBA (A9855) was acquired from Sigma-Aldrich (Bangalore, India). Estradiol (RM4682) was obtained from HiMedia Laboratories (Mumbai, India). The diagnostic kits for estimation of serum and urine calcium (CARZM 50), phosphorus (PHOSM 25), creatinine (CRE 100), alkaline phosphatase (ALP) (ALP SLR 25) were obtained from Lab-Care Diagnostics (Mumbai, India). NF-κB and TNF-α ELISA assay kits were purchased from Cayman Chemical (Ann Arbor, MI, USA). Other chemicals or reagents used in this study were acquired from local vendors and were of analytical grade.

### 2.2. Animals

Healthy adult Sprague Dawley (SD) female rats (weight; 200–250 g) were acquired from Zydus Research Centre (Ahmedabad, Gujarat, India). Rats were placed in controlled conditions (24 ± 2 °C temperature, 55 ± 5% humidity, 12 h/12 h light-dark cycle) and were access to food and water at the animal house of Institute of Pharmacy, Nirma University, Ahmedabad, India. A one-week acclimatization period was followed before starting the experiment. Animals were maintained and monitored for good health in accordance with test facility standard operating procedures. All experiments and protocols (IPS/PCOL/MPH/18/1011) were approved according to guidelines mentioned by the Committee for the Purpose of Control and Supervision of Experiments on Animals (CPCSEA), Ministry of Fisheries, Animal Husbandry and Dairying, India.

### 2.3. Experiment Design

#### 2.3.1. Osteoporosis Induction

The SD female rats were anesthetized by a single administration of ketamine (50 mg/kg) and xylazine (5 mg/kg) via intraperitoneal injection [24]. Adequate precautions were made to maintain the body temperature (37.5 ± 0.5 °C) of animals during the surgery. Ovariectomy was performed to induce osteoporosis by the procedure described by Taylor et al. [25]. Briefly, a midline dorsal bilateral skin incision was made, almost 3 cm in length, and almost midway between the central of the back and the base of the tail. Once the peritoneal cavity was reached, the ovary was identified and separated from surrounding fat. The blood vessels were then ligated and the joint connecting Fallopian tube and the uterine horn was cut using scissors and a blade to separate the ovary. The incision was sutured back and necessary post-operative care was taken to minimize animal suffering. The animals were allowed to move freely and had access to food and water after the surgery. The sham group of animals were operated as described above with regard to surgical site, dissection of peritoneal cavity, exposure of the ovaries, and closing. However, no ovariectomy was carried out in animals.

#### 2.3.2. Treatment Protocol

Female SD rats were separated into 4 groups, 6 rats each; sham-operated (1 mL/kg, orally in 0.5% *w/v* carboxymethyl cellulose), ovariectomized control (1 mL/kg, orally in 0.5% *w/v* carboxymethyl cellulose), ovariectomized treated with AKBA (35 mg/kg, orally in 0.5% *w/v* carboxymethyl cellulose), and ovariectomized treated with estradiol replacement therapy (0.05 mg/kg, intramuscularly in olive oil). AKBA was dispersed in 0.5% *w/v* carboxymethyl cellulose and was administered via gastric gavage. The dose of AKBA (35 mg/kg) was selected based on earlier reports [26] and our preliminary experiments. The estradiol solution was formulated by dissolving it in olive oil. The solution was injected in animals after 3rd day of ovariectomy and continued every other day. The same volume of vehicle was administered to both sham and ovariectomy groups. The treatment of AKBA, estradiol, and carboxymethyl cellulose to the respective group was continued for 42 days. The bodyweight of each female SD rats was checked weekly. On day 42, the animals were fasted for the entire night and were individually housed in metabolic cages for collection of urine up to 24 h. The urine was analyzed for determination of calcium, phosphorus, and creatinine levels. Further, the blood samples were collected after giving anesthesia to rats. Serum was separated from the collected blood samples by configuration and was stored (−80 °C) for further analysis of calcium, phosphorus, and ALP levels by colorimetric assay kits using an semi-automatic biochemical analyzer (Prietest Touch Plus; Robonik, Ambernath, Thane, India) [27]. NF-κB and TNF-α levels were estimated using ELISA assay kits as per the suppliers’ instruction. After the sample collection (blood and urine), the rats were euthanized, the uterine horn was isolated, removing the surrounding fat, and weighed immediately. The femur bone and lumbar vertebra were identified and then separated. The weight, thickness, and length of the femur bone and lumbar vertebra were measured using digital slide calipers (Mitutoyo South Asia, New Delhi, India). The length of the femur bone was determined by measuring the actual length between the proximal tip of the femur and the distal tip of the medial condyle [28]. Femur bone and lumbar vertebra were subjected to the measurement of BMD, compression test, and histopathological evaluation. Further, the femur bone and lumbar vertebra were ashed for the measurement of calcium content.

### 2.4. Estimation of Calcium Content in Femoral Bone and Lumbar Vertebral Ash

The calcium content was estimated for left femur and 4th lumbar vertebra. The bone marrow was removed from bones and were placed in fused silica crucibles separately, placed in a muffle furnace, and allowed to dry at a steady temperature (800 °C) for a period of 24 h. Following that, the assay for calcium level in the ash was performed. Bone ash (2 g) was weighed and dilute nitric acid was added to dissolve the ash completely. The solution was filtered and 1% ammonium hydroxide was added to the filtrate. Again, the solution was filtered and the filtrate was separated into two test tubes. Silver nitrate (1%) was included to one of the test tubes and to the other test tube 1% ammonium chloride and 1% potassium thiocyanate was added simultaneously and boiled. A white precipitate of calcium carbonate was observed, which was dried and weighed, and % calcium was calculated in ash [29].

### 2.5. Determination of BMD

BMD was measured for assessing bone loss in the animal using an X-ray bone densitometer. The total femur and vertebral BMD was measured using the procedure mentioned by Huang et al. [30]. Bone densitometry was carried out after administering anesthesia. The anesthetized animal was placed in a ventral position on the scan table. The scanning of rats were carried out by a dual-energy X-ray absorptiometry (OsteoSys, Aadi Medi Solutions, Jaipur, Rajasthan, India) to evaluate body BMD of rats.

### 2.6. Femur and Lumbar Vertebra Compression Test

The femur and lumbar vertebra were subjected to a compression test using method described by Shirwaikar et al. [31]. The separated femur was kept in an ordinal hardness tester and compressor (Instron, Chennai, Tamil Nadu, India). The pressure was applied and the force (kilopond) at which the bone fractured was noticed. Similarly, the fresh 4th lumbar vertebra was kept in the same digital hardness tester and compress unit, and the force required to break the bone was noticed.

### 2.7. Histopathological Analysis

A femur was harvested for bone histomorphometry analysis. The bone pieces were dipped in neutral buffered formalin (10%) and the decalcification was carried out by placing in ethylenediaminetetraacetic acid solution (10%). The decalcified bone pieces were fixed in paraffin wax, and were sectioned longitudinally with approximate bone thickness of 5 μm using HM 325 rotary microtome (SP 1600, Leica Biosystems, Nussloch, Germany). The bone sections were stained using hematoxylin and eosin [32]. The sections were visualized with a light microscope (Olympus CX23, Gurgaon, India).

### 2.8. Statistical Analysis

The data are expressed as mean ± standard error of the mean (SEM). The data were examined using one-way analysis of variance (ANOVA) followed by Tukey’s multiple comparison test using GraphPad Prism software version 5 (GraphPad, San Diego, CA, USA) [33]. The data are considered as statistically significant when the *p* value is < 5%.

## 3. Results

### 3.1. Effect of AKBA on Physical Parameters

#### 3.1.1. Effect of AKBA on the Body Weight and Uterus Weight

The results of ovariectomy group indicate significant surge in body weight together with significantly decreased uterine weight when compared with the sham-operated group. AKBA and estradiol-treated animals have shown a substantial (*p* < 0.01) rise in uterine weight in comparison to the disease control group, while there was an insignificant change in body weight by treatment with AKBA or estradiol compared to disease control rats (Table 1).

#### 3.1.2. Effect of AKBA on the Weight, Length, and Thickness of Femur Bone and Lumbar Vertebra

The ovariectomized control group showed a major decline in weight, length, and thickness of the femur bone and lumbar vertebra in comparison to the sham-operated rats. However, it is evident from Table 1 that the AKBA and estradiol-treated animals have shown a substantial rise in all these parameters when compared with the ovariectomized control group.

#### 3.1.3. Femur and Lumbar Vertebra Compression Test

Ovariectomized rats showed a significant reduction in hardness when compared with the sham-operated group. A significant (*p* < 0.001) improvement in compressibility was observed after treatment with AKBA and estradiol. Similar results (*p* < 0.05) were demonstrated for the lumbar vertebra compression test after treatment with AKBA and estradiol (Table 1).

#### 3.1.4. Effect of AKBA on Calcium Content of Femur Bone and Lumbar Vertebra

Calcium content in femur bones and lumbar vertebrae observed in Table 1 indicates significant (*p* < 0.001) decrease in the ovariectomized control group while there was significant improvement after treatment with AKBA and estradiol (Table 1).

#### 3.1.5. Effect of AKBA on BMD of Femur Bone and Lumbar Vertebra

The rats from the ovariectomized control group demonstrated a significant reduction in BMD of the femur bone and lumbar vertebra as compared to the sham-operated animals. However, the results in Table 1 indicate that the treated groups showed a statistically significant increase in the BMD values of the femur bone and lumbar vertebra by AKBA (*p* < 0.01) and estradiol (*p* < 0.05) (Table 1).

### 3.2. Effect of AKBA on Serum Biochemical Parameters in Ovariectomized Rats

**Figure 2:**
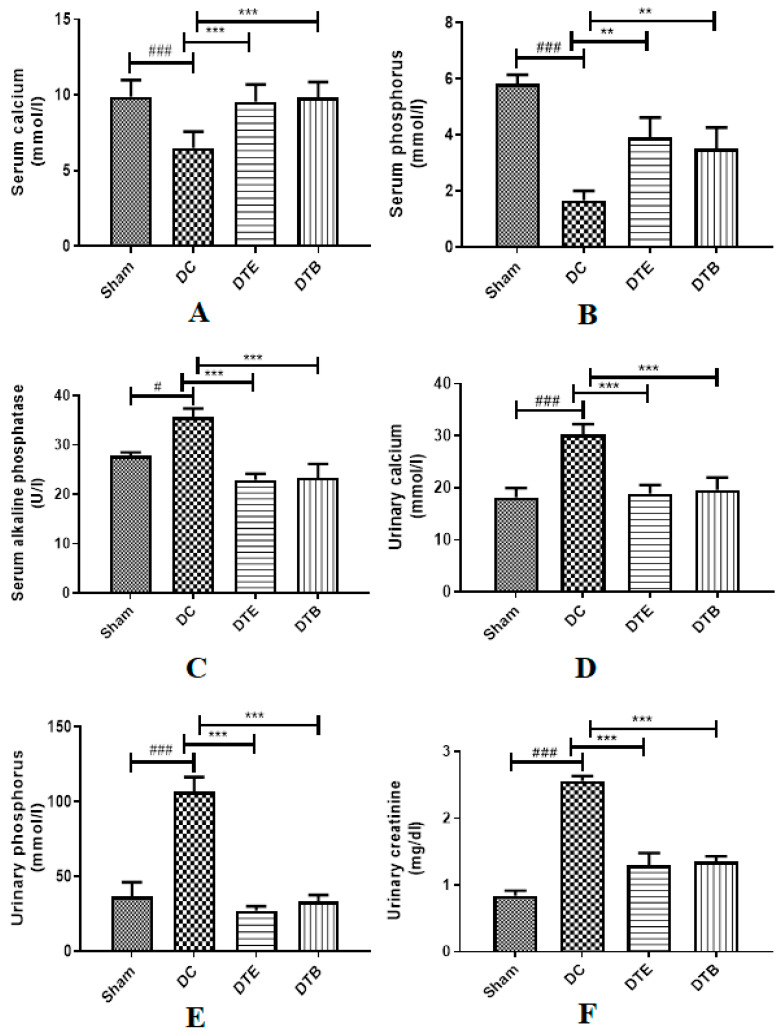
Effect of AKBA on serum as well as urine biochemical parameters in ovariectomized rats. (**A**) Serum calcium. (**B**) Serum phosphorus. (**C**) Serum alkaline phosphatase. (**D**) Urinary calcium. (**E**) Urine phosphorus. (**F**) Urinary creatinine. Data represented are mean ± SEM (*n* = 6) analyzed by one way ANOVA followed by Tukey’s multiple comparison test. ^#,###^ Statistically significant (*p* < 0.05 and *p* < 0.001, respectively) from sham control group. **^,^*** Statistically significant (*p* < 0.01 and *p* < 0.001, respectively) from disease control (DC) group. DTE: ovariectomized animals treated with estradiol (0.05 mg/kg); DTB: ovariectomized animals treated with AKBA (35 mg/kg). AKBA: acetyl-11-keto-β-boswellic acid.

The results of biochemical studies indicate that the serum calcium and phosphorus levels were significantly reduced in the ovariectomized control rat, when compared with the sham-operated group while serum ALP levels showed a significant increase as compared to the sham-operated rats. AKBA and estradiol-treated groups indicated substantial rise in serum calcium (*p* < 0.001) and phosphorus (*p* < 0.01) levels in comparison to ovariectomized rats. Serum ALP levels reduced considerably (*p* < 0.001) in AKBA and estradiol-treated groups (Figure 2A–C).

### 3.3. Effect of AKBA on Urine Biochemical Parameters in Ovariectomized Rats

The results of urine biochemical data indicate significantly higher (*p* < 0.001) calcium, phosphorus, and creatinine levels in the ovariectomized control group when compared with the sham-operated group. However, a significant (*p* < 0.001) decrease in the levels of these parameters was seen in AKBA and estradiol-treated rats in comparison to the ovariectomized control rats (Figure 2D–F).

### 3.4. Effect of AKBA on NF-κB and TNF-α Expressions

**Figure 3:**
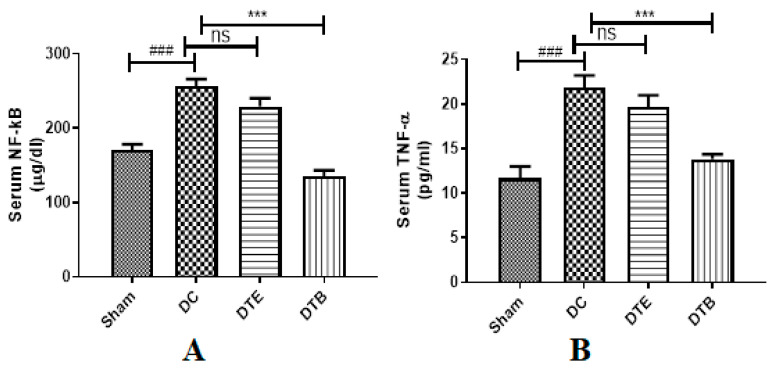
Effect of AKBA on serum inflammatory markers in ovariectomized rats. (**A**) Serum NF-κB and (**B**) Serum TNF-α. Data represented as mean ± SEM (*n* = 6) examined by one way ANOVA followed by Tukey’s multiple comparison test. ^###^ Statistically significant (*p* < 0.001) from sham control group. *** Statistically significant (*p* < 0.001) from disease control (DC) group. DTE: ovariectomized animals treated with estradiol (0.05 mg/kg); DTB: ovariectomized animals treated with AKBA (35 mg/kg). AKBA: acetyl-11-keto-β-boswellic acid; ns: non-significant.

The ovariectomized control animals have shown substantial (*p* < 0.001) increase in the level of NF-κB and TNF-α in comparison to the sham-operated rats. The AKBA-treated rats have shown major (*p* < 0.001) reduction in NF-κB and TNF-α levels in comparison to the ovariectomized control rats. At the same time, the estradiol-treated group did not produce any major changes in both tested parameters (Figure 3A,B).

### 3.5. Effect of AKBA on Histopathological Changes of Ovariectomized Rats

**Figure 4:**
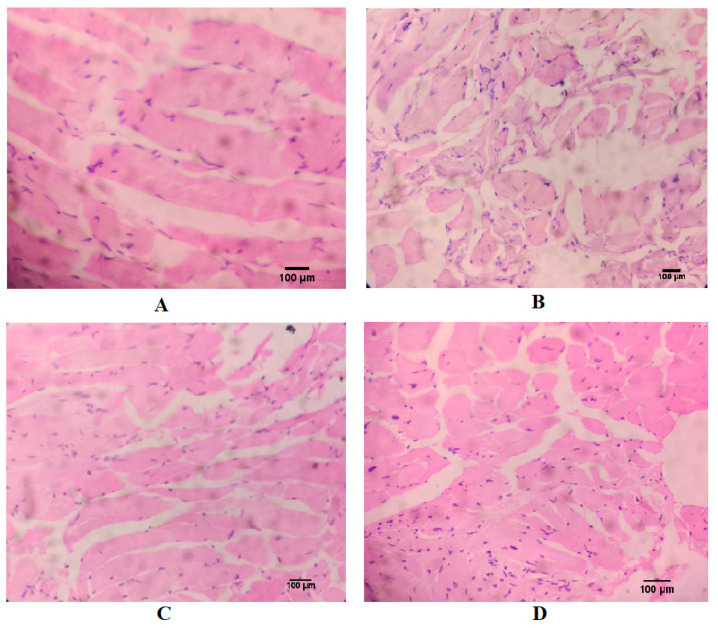
Effect of AKBA on histopathological changes in ovariectomized rats. (**A**) Sham control. (**B**) Ovariectomized control. (**C**) Ovariectomized animals treated with estradiol (0.05 mg/kg). (**D**) Ovariectomized animals treated with AKBA (35 mg/kg). AKBA: acetyl-11-keto-β-boswellic acid.

In histopathological examination, sham control showed a normal femur bone structure with lacuna and many lamellae. The ovariectomized control group showed porosity with presence of reduced intertrabecular spaces. In ovariectomized animals treated with AKBA, the bone structure was found to be regular with intertrabecular spaces and free from any destruction or porosity in the femur bone. A similar histoarchitecture was also observed in the estradiol-treated group (Figure 4).

## 4. Discussion

The current study evaluated the effect of AKBA on osteoporosis induced by ovariectomy. It is well known that bone loss in osteoporosis is commonly related to estrogen deficiency during menopause. Estrogen deficiency results in an increase in plasma calcium levels due to increased bone resorption [34]. It is also known that hormone or estrogen replacement therapy (HRT/ERT) is generally considered one of the possible options to prevent and treat postmenopausal osteoporosis, though it generally leads to various adverse effects, such as breast cancer, uterine cancer, and thromboembolic disease [34,35]. On the other hand, women administered with ERT have higher chance of uterine cancer, and is more at risk of breast cancer when administered for long term such as more than 15 years, as compared to those not taking ERT [36]. In this context, discovering alternative therapeutic moieties for the management of osteoporosis is the need of the hour [37]. In the current study, the choice of AKBA was based on the various therapeutic potential described in the literature [10,12,13,14,15]. The literature also signifies that the boswellic acid is safe and is free from toxicity as no pathological changes were noticed in animals when assessed for subacute toxicity study where biochemical, hematological and histopathological parameters were determined at a dose up to 1000 mg/kg [38]. Moreover, the LD_50_ of boswellic acid was reported to be >2 g/kg [39].

Literature also suggests that the ovariectomized rat is considered as an ideal preclinical animal model, which resembles the chief clinical feature of the estrogen deficiency in humans and the effect of antiosteoporotic agents. The ovariectomized rat is considered a suitable animal model for the study of menopausal osteoporosis because of the many similarities in their pathological and clinical features. Bone loss is quick after the onset of estrogen deficit, and it is characterized by an augmented bone turnover [40].

Ovariectomy causes body weight gain in rats [41]. Ovariectomized rats reported gaining fat, specifically visceral fat [42]. Estradiol was reported to restore central leptin sensitivity and alter body fat distribution in ovariectomized rats. However, we found an insignificant decrease in the body weight gain in ovariectomized rats administered with either AKBA or estradiol.

The bone loss in relation to uterine weight was noticed during the study. Ovariectomy causes atrophy of the uterus [43]. A significant reduction in uterine weight was observed in ovariectomized rats, indicating uterus atrophy. This effect was prevented by the administration of AKBA and estradiol. Indeed, the effect of AKBA was found to be greater than estradiol.

Biochemical parameters of bone resorption have been extensively used as a tool of investigation to evaluate the effects of various drugs on bone restoration. Menopause is accompanying with amplified renal elimination of calcium and reduced calcium absorption from the intestine and has negative calcium balance [44]. Reports also showed that bone turnover in the ovariectomized rat is associated with a significant increase in urinary calcium and phosphorous levels [45]. In the current study, it was noticed that the serum calcium and phosphorus level decreased in ovariectomized rats. Previous reports suggest that the activated estrogen receptor stimulates endocytotic absorption of calcium and phosphorus and its transportation in the duodenal cells [46,47]. Treatment with AKBA showed increased calcium absorption, which is evident from increased levels of calcium and phosphorus in the serum of ovariectomized rats. Similarly, a higher amount of calcium and phosphorous was lost through the urine, which also points to the fact that greater bone loss has occurred in the ovariectomized rats. The clearance of calcium and phosphorus in urine was drastically decreased by the administration of AKBA and estradiol, and a possible explanation for these findings could be the estrogenic effect of AKBA. Higher ALP in serum generally happens in diseases of bone and liver [48]. An increase in the level of ALP was observed in ovariectomized rats. Treatment with AKBA showed a decrease in ALP activity in ovariectomized rats. These results suggested that AKBA decreased bone resorption and increased mobilization of minerals in bones. It is also reported that the ovariectomy can promote kidney disease and has been demonstrated in various animal models [49,50]. In the current study, kidney function tests demonstrated that the AKBA exhibited reduced kidney damage, which was produced due to ovariectomy.

Ovariectomy caused bone loss was determined by decreased bone length, weight, and thickness of femur bone and lumbar vertebra [40]. The results showed that the administration of AKBA for 42 days was capable to increase the weight, length, and thickness of femur bone and lumbar vertebra. This effect of AKBA was almost equal to the effect of estradiol on the same parameters in femur bone and lumbar vertebra. Furthermore, the administration of AKBA could restore the decline of femoral ash calcium content to a visible level, establishing its positive effect on calcium absorption in ovariectomized rats.

Sex hormone deficiency in animals due to ovariectomy leads to a decrease in density, hardness, and stiffness of bones. Augmented loss of bone occurs in females after menopause. Both osteoporosis and menopause share numerous analogous characteristics [40]. Estrogen receptor agonist improves calcium balance in patients with postmenopausal osteoporosis [34]. X-ray bone densitometer is mainly used to determine total BMD in human beings as well as in animal experiments. It helps for initial quantitative analysis for bone and can rapidly give information about the quality of bone specimens. Results obtained in our study signified that ovariectomy decreased BMD and bone hardness in untreated animals. AKBA has also shown a significant improvement in the mechanical properties of the femur bone and lumbar vertebra. Thus, AKBA, by increasing serum calcium levels involved in the mobilization of bone minerals, as observed from the results of BMD, can increase bone hardness.

The majority of the inflammatory effects of TNF-α are through stimulation of NF-κB [51] which has also been shown to suppress osteoblastic activity [52,53]. RANKL has been shown to facilitate osteoclastogenesis mediated through NF-κB signaling pathway [22,54]. Previous reports suggest that AKBA produces a suppression of TNF-α formation via interference with the NF-κB activation pathway, and consequently, this suppression contributes to its anti-inflammatory effectiveness [55]. Thus, the inhibition of TNF-α abrogated RANKL-induced osteoclastogenesis by a downregulation of NF-κB regulated gene expression. In the present study, a significant increase in serum NF-κB and TNF-α levels was observed in ovariectomized rats, which is an indicator of increased osteoclastic activity that accounts for osteoporosis. AKBA showed a significant reduction in both inflammatory markers [10,56] whereas surprisingly estradiol did not show any significant change in these markers. Thus, our results indicate that AKBA produced its anti-osteoporotic effect via the inhibition of NF-κB-regulated gene expression.

Estrogen receptor activation in osteoblasts stimulates expression of special proteins and growth factors, which is responsible for bone formation [57]. In the current study, histopathological examinations reveal that the section of femurs of ovariectomized rats showed porosity with the presence of reduced intertrabecular spaces. On the other hand, the group treated with the AKBA showed normal bone structure, intertrabecular spaces without any damage or porosity in the bone, which is also similar to the estradiol-treated group. The results indicate the possible protective effect of AKBA that might be due to increased bone mineralization with a reduction in bone loss.

## 5. Conclusions

Thus, we can conclude that treatment with AKBA significantly reduced bone loss, which was evident from the measured physical and biochemical parameters. Moreover, histopathological investigation showed restoration of the normal architecture of bone. AKBA showed good antiosteoporotic activity in comparison with the standard drug estradiol. Further, the present finding strongly suggests that the antiosteoporotic activity of AKBA may be mediated through its inhibition of the NF-κB-induced TNF-α signaling pathway, which consequently substantiates its possible use in the management of postmenopausal osteoporosis.

## Figures and Tables

**Table 1:** Effect of acetyl-11-keto-β-boswellic acid (AKBA) on physical parameters in ovariectomized rats.

Parameter	Sham	DC	DTE	DTB
Body weight gain (g)	24.7 ± 1.88	45.8 ± 2.95 ^###^	40.6 ± 1.43 ^###^	39.5 ± 1.97 ^###^
Weight of uterus (g)	0.64 ± 0.07	0.45 ± 0.01 ^#^	0.68 ± 0.05 **	0.74 ± 0.03 **
Weight of the femur bone (g)	0.59 ± 0.03	0.33 ± 0.01 ^###^	0.49 ± 0.03 **	0.49 ± 0.03 **
Length of femur bone (mm)	23.1 ± 0.51	20.3 ± 0.15 ^##^	22.5 ± 0.51 **	22.9 ± 0.46 **
Thickness of femur bone (mm)	5.18 ± 0.15	4.27 ± 0.05 ^###^	5.19 ± 0.06 ^##^***	5.29 ± 0.18 ^#^***
Hardness of femur bone (kilopond)	4.98 ± 0.18	2.69 ± 0.12 ^###^	5.14 ± 0.06 ***	5.31 ± 0.25 ***
% Calcium in femur bone	1.33 ± 0.07	0.41 ± 0.14 ^###^	1.02 ± 0.06 **	1.05 ± 0.14 **
BMD of femur bone (mg/mm^2^)	178 ± 8.36	118 ± 9.13 ^###^	150 ± 8.67 *	162 ± 5.55 **
Weight of the lumbar vertebra (g)	0.33 ± 0.03	0.18 ± 0.01 ^##^	0.30 ± 0.03 *	0.32 ± 0.04 *
Length of lumbar vertebra (mm)	8.04 ± 0.44	6.20 ± 0.40 ^##^	7.55 ± 0.23 *	7.96 ± 0.19 **
Thickness of lumbar vertebra (mm)	3.89 ± 0.13	2.25 ± 0.12 ^###^	2.93 ± 0.16 ^##^*	3.20 ± 0.20 ^#^**
Hardness of lumbar vertebra (kilopond)	12.9 ± 0.56	9.47 ± 0.73 ^##^	11.9 ± 0.44 *	12.4 ± 0.58 *
% Calcium in lumbar vertebra	1.13 ± 0.09	0.49 ± 0.11 ^###^	0.91 ± 0.06 *	1.03 ± 0.10 **
BMD of lumbar vertebra (mg/mm^2^)	106 ± 5.28	71.2 ± 7.56 ^##^	96.0 ± 4.54 *	105 ± 5.44 **

Data represented are mean ± SEM (*n* = 6) analyzed by one way ANOVA followed by Tukey’s multiple comparison test. ^#,##,###^ Statistically significant (*p* < 0.05, *p* < 0.01, and *p* < 0.001, respectively) from sham control group. *^,^**^,^*** Statistically significant (*p* < 0.05, *p* < 0.01, and *p* < 0.001, respectively) from disease control (DC) group. DTE: ovariectomized animals treated with estradiol (0.05 mg/kg); DTB: ovariectomized animals treated with AKBA (35 mg/kg). BMD: bone mineral density.
